# Spatio-temporal analysis of the role of climate in inter-annual variation of malaria incidence in Zimbabwe

**DOI:** 10.1186/1476-072X-5-20

**Published:** 2006-05-15

**Authors:** Musawenkoi LH Mabaso, Penelope Vounatsou, Stanely Midzi, Joaquim Da Silva, Thomas Smith

**Affiliations:** 1 Malaria Research Lead Programme, Medical Research Council, P.O. Box 70380, Overport 4067, Durban, South Africa; 2 Public Health and Epidemiology, Swiss Tropical Institute, Socinstrasse 57, P.O. Box CH-4002, Basel, Switzerland; 3 National Malaria Control Programme, Ministry of Health and Welfare, P.O. Box CY1122, Causeway, Harare, Zimbabwe; 4 World Health Organization Southern Africa Inter-Country Programme for Malaria Control, P.O. Box CY348, Causeway, Harare, Zimbabwe

## Abstract

**Background:**

On the fringes of endemic zones climate is a major determinant of inter-annual variation in malaria incidence. Quantitative description of the space-time effect of this association has practical implications for the development of operational malaria early warning system (MEWS) and malaria control. We used Bayesian negative binomial models for spatio-temporal analysis of the relationship between annual malaria incidence and selected climatic covariates at a district level in Zimbabwe from 1988–1999.

**Results:**

Considerable inter-annual variations were observed in the timing and intensity of malaria incidence. Annual mean values of average temperature, rainfall and vapour pressure were strong positive predictors of increased annual incidence whereas maximum and minimum temperature had the opposite effects. Our modelling approach adjusted for unmeasured space-time varying risk factors and showed that while year to year variation in malaria incidence is driven mainly by climate, the resultant spatial risk pattern may to large extent be influenced by other risk factors except during high and low risk years following the occurrence of extremely wet and dry conditions, respectively.

**Conclusion:**

Our model revealed a spatially varying risk pattern that is not attributable only to climate. We postulate that only years characterized by extreme climatic conditions may be important for developing climate based MEWS and for delineating areas prone to climate driven epidemics. However, the predictive value of climatic risk factors identified in this study still needs to be evaluated.

## Background

The risk of malaria infection varies widely according geographic region, season and year [1,2]. On the fringes of endemic zones, particularly at the southernmost latitudes in southern Africa, across arid regions of northern Africa and among the highlands of east and central horn of Africa climate is a major determinant of seasonal and inter-annual (year to year) variation in malaria transmission [3].

In Southern Africa annual variation in climatic conditions and associated changes in malaria infection affect the timing and intensity of malaria incidence. This has an impact on the effectiveness of interventions [4]. As a result there is a need for the development of climate-based malaria early warning systems (MEWS) capable of predicting seasonal to inter-annual variations with a lead time that allows health authorities to respond in a timely manner with preparatory/preventative measures [5,6]. However, despite the fact that climate data are often used to account for spatial, seasonal and inter-annual variation in malaria risk in Africa, there is often little or no consensus about the relative importance and predictive value of different factors involved [7]. The disagreements seem to stem from differences in perspective and methods used [8].

In Southern Africa, few studies or models of the relationship between malaria and climatic factors have been published. In a recent meeting of the Southern African Inter-Country Programme on Malaria Control (SAMC) a number of countries acknowledged having poor empirical basis on which to develop and test climate-based early warning and detection indicators [4]. Existing models include a non-spatial model for Zimbabwe which identified temperature as the main determinant of increased malaria risk years [9]. Spatial and temporal models which used both temperature and rainfall for analysis and mapping of malaria risk in KwaZulu-Natal, South Africa [10,11]. An exploratory analysis of 30 years worth of data in the epidemic prone area of KwaZulu-Natal, South Africa, which showed that certain aspects of climate appear to drive inter-annual variation of malaria incidence but not its overall level [12]. In a recent study Thomson et al [13] showed that rainfall and sea surface temperature (SST) have a potential for application in the development of seasonal forecasts [13]. We also recently developed a space-time seasonality model based on the relationship between monthly clinical malaria case data and environmental factors in Zimbabwe [14].

Year to year predictability of malaria incidence still remains a challenge, and more work is required before malaria climate based forecasting models can realize their full potential in the region. In this study we use Bayesian spatio-temporal analysis to describe year to year variation of malaria incidence data from Zimbabwe in relation to variation in climatic risk factors to enhance our ability of developing an operational MEWS and determine areas prone to climate-driven epidemics.

## Methods

### Setting

In common with Angola, Namibia, Botswana, Zambia, Mozambique and South Africa Zimbabwe lies at the southern limits of malaria distribution in Africa. Malaria remains a major cause of mortality and morbidity despite more than four decades of sustained national control programme [15]. Moreover, as a result of reduced level of transmission there is propensity for malaria epidemics unless adequately controlled or prevented. Overall, about 45–50% of the 12.5 million people of Zimbabwe are at risk of malaria. In 1998, it was estimated that approximately 8% of all deaths and 12% of all outpatient cases were due to malaria . Recently, substantial socio-economic changes have further compromised the malaria control programme [4].

Climate is another major factor that determines the extent of malaria transmission in Zimbabwe, and its variability may work with or against efforts to bring malaria under control [16]. The most important factors governing malaria epidemiology in the country are season, altitude, and associated rainfall and temperature changes [14,16-18]. Malaria is found mainly within the low and mid altitude zones and rarely at higher altitude. However, this can vary tremendously from one year to another.

### Covariate data

We used normalized difference vegetation index (NDVI) available between 1988 and 1999 from Advanced Very High Resolution Radiometer (AVHRR) sensor onboard the National Oceanic and Atmospheric Administration (NOAA) satellite . We also used mean annual values of rainfall, vapour pressure, minimum, maximum and mean temperature obtained for each district and year for the 12 year period. These were obtained from the climate research unit (CRU) climate surfaces derived from interpolated weather station data as a function of latitude, longitude, and elevation using thin-plate splines [19].

### Malaria data

In Zimbabwe malaria is a notifiable disease and records from hospitals and clinics are compiled at a district, provincial and national levels to describe the malaria situation and trends. We used annual clinical malaria case data for children under the age of five reported in 58 districts covering the whole country between 1988 and 1999 [20]. This is the highest risk group, with relatively little protective immunity and is therefore expected to be more sensitive to changes in malaria transmission. The data included both microscopically confirmed and unconfirmed clinically diagnosed cases. District population projections based on the 1982 and 1992 census were used to calculate incidence rate per 1000 person years [21].

### Analysis

We used the annual proportion of monthly malaria cases and Markham's seasonality index [22,23] to display between-year variation in the data. The seasonality index has been described in detail elsewhere [14]. Briefly, this method calculates the seasonal concentration of the malaria case load and the peak month in a given year.

A preliminary negative binomial regression analysis was carried out in STATA 9.0 (Stata Corp., College Station, TX, USA) to assess the relationship between annual malaria incidence and annual values of each climatic covariate. Thereafter, Bayesian negative binomial models were fitted in WinBUGS [24] to examine the association between inter-annual variation in malaria incidence and a combination of climatic covariates selected from the preliminary analysis (see the appendix for more details). Basically, spatial random effects were used at a district level to take into account spatial correlation present in the data. Temporal random effects were also used at yearly intervals to account for temporal correlation. Spatial correlation was incorporated by assuming a conditional autoregressive (CAR) process in the random effects. A first order autoregressive process was applied for temporal random effects [25].

Markov Chain Monte Carlo simulation (MCMC) was applied to estimate model parameters [26]. After the initial burn-in of 5000 the number of iterations thereafter depended on convergence which was assessed using ergodic averages. After convergence a final sample of 5000 was collected to obtain summaries of the posterior distribution of the parameters. The Deviance Information Criterion (DIC) [27] was used for the comparison of model fit. Small values of DIC indicate superior model fit. Model estimates were exponentiated to represent incidence rate ratios (IRR), that is, per unit change in incidence for each covariate.

## Results

**Figure 1:**
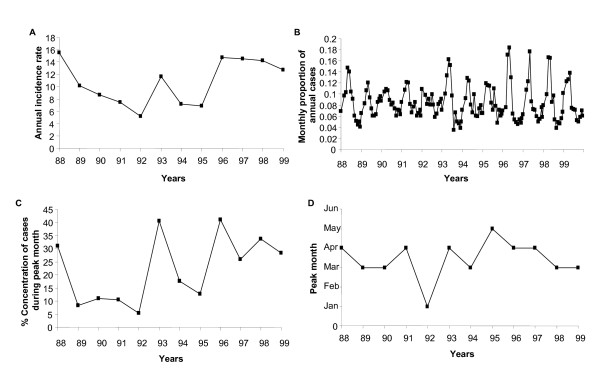
(A) Annual malaria incidence rate (cases per 1000 person years) (B) proportion of annual monthly cases (C) percentage concentration of malaria case load during the peak transmission month and (D) peak month during the malaria transmission season in Zimbabwe from 1988–1999.

Figure 1A–D shows that malaria transmission in Zimbabwe is characterized by considerable between and within year variations. The highest malaria incidence recorded during the 12 year period was in 1988 with 15.5 cases per 1000 person years and the lowest was in 1992 with 5.2 cases per 1000 person years (Figure 1A). In addition since 1996, there is a rising trend in annual incidence with reported malaria cases remaining at high levels. The intensity and timing of the seasonal peak also varies from year to year (Figure 1B &1D). From 1988 to 1999 the peak month fluctuated between March and April with the exception of 1992 and 1995 which were characterized by peaks in January and May respectively.

**Figure 2:**
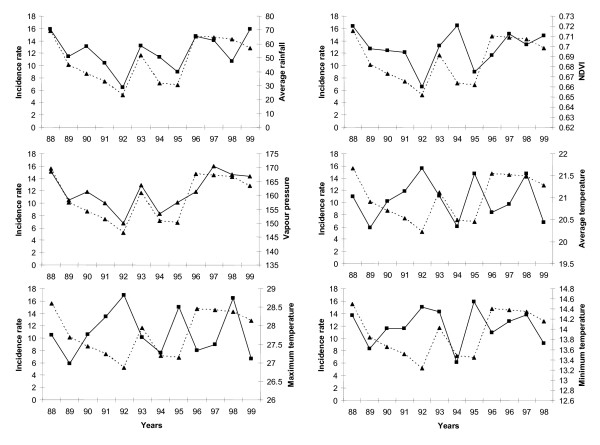
Inter-annual variations in malaria incidence rate (cases per 1000 person years), rainfall (mm), vapour pressure (hPa), NDVI (Normalized Difference Vegetation Index), average, maximum and minimum temperatures (°C) in Zimbabwe between 1988 and 1999.

High annual malaria incidence coincide with high rainfall and relatively warm conditions while low incidence years coincide only with low rainfall (Figure 2). Vapour pressure and NDVI follow the rainfall pattern. Temperature derived covariates seem to be important only in the presence of sufficient rainfall. The intensity and timing of the seasonal peak in each year appears to follow variability in rainfall.

In the bivariate analysis all selected covariates showed a significant relationship (P < 0.001) with malaria incidence (Table 1). All models (Table 2) indicated that mean annual temperature, rainfall, vapour pressure and NDVI were strong positive predictors of increased annual incidence rate in contrast maximum and minimum temperature had a reducing effect. However, in the spatial model (model 2) rainfall had no significant effect and in the spatio-temporal model (Model 3) only NDVI was not significant. Model comparison showed that the spatial-temporal model had a small DIC value and therefore was the best fitting model. This model had large spatially correlated random effects.

**Table 1:** Bivariate analysis of the relationship between annual malaria incidence and climatic covariates fitted using negative binomial regression.

**Covariates**	**Coefficients**	**SE**	**95% CI**	**P-value**
Mean temperature (°C)	0.295	0.024	0.248, 0.341	< 0.001
Maximum temperature (°C)	0.149	0.021	0.107, 0.189	< 0.001
Minimum temperature (°C)	0.439	0.024	0.391, 0.487	< 0.001
Vapour pressure (hPa)	0.046	0.003	0.040, 0.051	< 0.001
NDVI	0.654	0.127	0.405, 0.903	< 0.001
Rainfall (mm)	0.021	0.002	0.016, 0.026	< 0.001

SE – standard error; CI – confidence intervals; NDVI – normalized difference vegetation index

**Table 2:** Modelled estimates of the effects of climatic covariates on malaria incidence in the districts of Zimbabwe, including spatial and temporal variance. The smaller value of DIC indicates a better fitting model.

**Covariates**	**Non spatial Model**	**Spatial Model**	**Spatial-temporal model**
	IRR (95% CI)	IRR (95% CI)	IRR (95% CI)
Mean temperature (°C)	5.332 (4.700, 5.885)	6.533 (4.251, 8.812)	7.634 (6.890, 8.349)
Maximum temperature (°C)	0.440 (0.414, 0.485)	0.363 (0.306, 0.446)	0.291 (0.272, 0.322)
Minimum temperature (°C)	0.700 (0.657, 0.752)	0.479 (0.357, 0.623)	0.500 (0.412, 0.581)
Vapour pressure (hPa)	1.003 (0.998, 1.008)	1.036 (1.020, 1.050)	1.018 (1.005, 1.028)
NDVI	2.700 (2.267, 3.132)	1.478 (1.011, 2.256)	1.375 (0.913, 1.701)
Rainfall (mm)	1.017 (1.012, 1.021)	1.005 (0.999, 1.011)	1.006 (1.000, 1.012)
Spatial variation ()		1.346 (1.078, 1.673)	18.620 (15.280, 22.710)
Temporal variation ()			0.004 (0.001, 0.010)
DIC	8414.270	8113.280	7912.610

NDVI – normalized difference vegetation index; DIC – deviance information criterion; IRR – incidence rate ratio; CI – credible intervals

**Figure 3:**
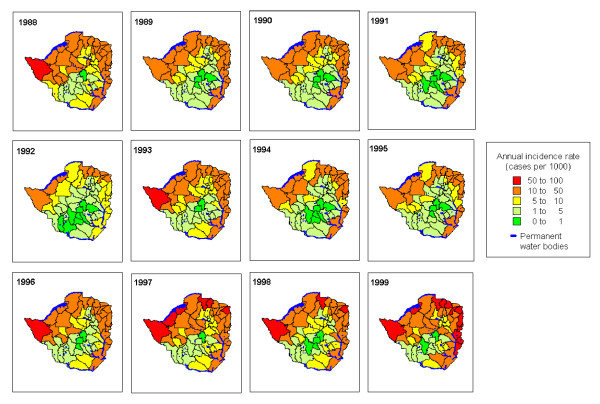
Geographic distribution of smoothed malaria incidence (cases per 1000 person years) by year between 1988 and 1999 in Zimbabwe from a spatial-temporal model.

Figure 3 show differences in the spatial pattern of modelled malaria incidence during the 12 year study period. In 1988, 1993 and 1996 onwards high to moderate incidence rates were more widespread with the highest incidence rates in the north western and eastern part of the country. In 1991 to 1992 and 1994 to 1995 incidence rates were predominantly moderate to very low levels across the country with pockets of high incidence rates in the districts situated along of the Zambezi river system in the north western part and on the border with Mozambique in the eastern part as well as along the Limpopo river system in the south eastern part.

## Discussion

Our observations confirm that malaria transmission in Zimbabwe is characterized by substantial inter-annual variation. Incidence rates show a rising, albeit fluctuating, trend with reported cases remaining relatively high after 1996 (Figure 1). The intensity and timing of seasonal transmission also varies from year to year. The highest and lowest risk years were recorded during one of the most wet and severe drought periods in Zimbabwe, respectively [28]. From a regional perspective 1988, 1993, 1996 and 1997 were the most serious epidemic years [28].

Besides the limitation imposed by the effect of misdiagnosis as a consequence of unconfirmed clinically diagnosed cases, which would probably be to smooth over differences since this is not influenced by covariates. There are multiple explanations for the observed trend and it is difficult to identify true causes [8]. In general, there is more support for non-climatic explanations of recent trends especially in the fringe areas of endemic zones in Africa. These include deterioration of malaria control efforts, development of drug and insecticide resistance and a rise in co-infection with HIV/AIDS [8,29-31]. In Zimbabwe, none of these have been adequately quantified for inclusion into the modelling framework. Hence our modelling approach adjusts for unmeasured spatially and temporally structured sources of variation while investigating the association between annual malaria incidence and climatic covariates. However, the large residual spatial variation observed in the spatial-temporal model suggests that there are other important covariates not accounted for in the analysis that could explain most of the spatial variation in malaria incidence. Most of the temporal variation in the data appears to be explained by the selected covariates.

Our results showed that annual mean values of temperature, rainfall and vapour pressure are strong positive predictors of increased annual malaria incidence. Annual minimum and maximum temperature had the opposite effects probably due to fact that in an average annual cycle these climatic covariates are associated with the cold dry period (June-August) and hot dry period (August to October), respectively, both of which have low transmission [14]. The association between vapour pressure (a measure of humidity) and annual incidence reflect the importance of the interaction between rainfall and temperature which modulates the ambient air humidity which in turn affect the survival and activity of *Anopheles *mosquitoes [32,33].

Furthermore, the spatio-temporal model (Table 2) showed that while NDVI, a surrogate for the response of vegetation to rainfall, appears to have a positive effect, it had no statistically significant association with annual malaria incidence. This may be due to the fact that vegetation greenness is to a large extent dependant on the amount of rainfall available in a given year [34,35]. Moreover, total annual rainfall in Zimbabwe is characterized by strong variability [16]. Hence, rainfall is a stronger predictor of malaria incidence at an inter-annual time scale.

The modelled spatial pattern showed that areas in the low lying north-western and south-eastern part of the country have high but less variable incidence, whereas areas of lower incidence in the middle to highland areas showed greater year to year variability. Eco-epidemiological conditions in these areas determine the stability of between year variation in malaria transmission [16,34,36]. Our analysis therefore corroborates the fact that year to year variation in malaria incidence in Zimbabwe is driven mainly by climatic covariates, and further demonstrates that resultant incidence in a given area may also be a function of other unmeasured risk factors. The spatial effect of climatic conditions during the study period appears to be more evident only during the much reported drought years from 1990–1992 and following heavy rainfall in 1998 [28].

In our previous analysis optimum ranges of climatic risk factors identified in the present study were also significantly associated with malaria transmission at a seasonal time scale [14]. It is likely therefore that increase in mean annual temperature, rainfall and humidity is linked to within year changes in average climatic conditions and in turn lead to changes in the full annual cycle of malaria transmission. The magnitude of which probably varies from one year another. For example, Freeman and Bradley [9] found that higher than average annual mean monthly temperatures during the critical period of malaria transmission (April and September) in the previous year were associated with an increase in the severity of malaria in the following year in some areas. This becomes more pronounced and widespread if both extremes of warm and wet conditions coincide and vice versa as observed during high and low incidence years (Figure 2 and 3). We deduce that extreme climatic events associated with covariates identified in this study may be useful for developing climate based malaria forecasting models operational at both seasonal and inter-annual time scales.

In conclusion, our modelling approach adjusted for unobserved spatial and temporal varying risk factors, and showed that while inter-annual variation in malaria incidence is driven mainly by climatic conditions, the resultant spatially varying risk pattern may also be influenced by other risk factors. Nevertheless, high and low incidence years following the occurrence of extreme climatic conditions may be useful for developing climate based MEWS and for delineating areas prone to climate driven epidemics. However, the predictive value of climatic risk factors identified in this study still needs to be evaluated.

## Abbreviations

AVHRR

Advanced Very High Resolution Radiometer

CRU

Climate research unit

CAR

Conditional autoregressive

DIC

Deviance Information Criterion

MEWS

Malaria early warning system

MCMC

Markov Chain Monte Carlo simulation

NOAA

National Oceanic and Atmospheric Administration

NDVI

Normalized Difference Vegetation Index

SST

Sea surface temperature

SAMC

Southern African Inter-Country Programme on Malaria Control

## Competing interests

The authors of this paper do not have any commercial interest or other associations which pose a conflict of interest with this paper.

## Authors' contributions

MLHM conceptualized, analyzed and drafted the manuscript. PV and TM participated in the conception, analysis and drafting of the manuscript. SM and JDS critically reviewed the manuscript.

## Appendix: statistical model

We assumed that the observed counts of malaria cases *Y*_*it *_in district *i *(*i *= 1,..., 58) and year *t *(1988–1999) follow a negative binomial distribution with parameters *p*_*it *_and *r*, that is, Y_*it *_~ NB (p_it_, r), where p_it _relates to average number of cases via the formula (*μ*_*it*_) = *p*_*it*_/*r *and r is the overdispersion parameter. We modelled average number of new cases (*μ*_*it*_) as a function of potential risk factors as follows:

(1) log(*μ*_*it*_) = log *N*_*it *_+ *α *+ ***β ***    (non-spatial model)

(2) log(*μ*_*it*_) = log *N*_*it *_+ *α *+ ***β ***+ *ø*_*i *_    (spatial model)

(3) log(*μ*_*it*_) = log *N*_*it *_+ *α *+ ***β ***+ *ø*_*i *_+ *ω*_*it *_    (spatio-temporal model)

*N*_*it *_denotes population at risk in district *i *and year *t*, *α *is the incidence rate when all covariates have zero value, ***X***_*it *_is a vector of climatic covariate effect in district *i *and year *t*, ***β ***a vector of the regression coefficients, *ø*_*i *_is the spatial random effect for district *i *and *ω*_*it *_is the temporal random effect for year *t *and district *i*. District specific random effects were modelled via a conditional autoregressive (CAR) process, which implies that each *φ*_*i *_conditional on the neighbour *φ*_*j *_follows a normal distribution with mean equal to the average of neighbouring *φ*_*j *_and variance inversely proportional to the number of neighbours *n*_*i*_, that is, *ø*_*i*_|*ø*_*j*_, *j *neighbouring of *i *~ Normal () where *γ *is a parameter that quantifies the amount of spatial correlation present in the data and  measures the spatial variance. The temporal effects were modelled by first order autoregressive process with temporal variance , which allows correlation between consecutive time periods for each district and year. We assumed inverse gamma hyper-prior distributions for the variance parameters of the spatial and temporal random effects, non-informative Uniform prior distributions *U*(-∞, ∞) for the regression coefficients ***β***, and a Uniform distribution *U*(*a*, *b*) for *γ*, with limits *a *and *b *specified as described in Gelfand and Vounatsou [37].
